# Health Staff Perceptions of Hate Violence in Spain

**DOI:** 10.3390/ijerph18147591

**Published:** 2021-07-16

**Authors:** Patricia Fernández de Castro, Natalia Hipólito Ruiz, Eduardo Díaz Herráiz

**Affiliations:** Faculty of Social Sciences, University of Castilla-La Mancha, 45600 Talavera de la Reina, Spain; patricia.fernandez@uclm.es (P.F.d.C.); natalia.hipolito@uclm.es (N.H.R.)

**Keywords:** hate violence, health personnel perceptions, health service detection, public health

## Abstract

The aim of this study of Spanish health personnel is to determine their level of knowledge about hate violence and their relevance in detecting victims of hate violence and clarifying the magnitude of the phenomenon. An exploratory study with a descriptive, observational, and cross-sectional design was conducted, with incidental non-probabilistic sampling and an ad hoc questionnaire to health professionals in three Autonomous Communities of Spain. Our results indicate a general lack of knowledge about hate violence by health staff who acknowledged that they do not have specific training for hate violence victims’ care, although most staff had attended to some cases of hate violence in the last year. No significant differences were found among healthcare services, professionals, training, or Autonomous Communities, which indicated a generalized lack of training and specific tools that was common in the different health services and in different Autonomous Communities in Spain. The health services that reported most cases of hate violence ex officio were those in which the professionals had more training and knowledge and in which there were specific protocols on hate violence. In conclusion, the health system constitutes “the gateway” to the care, promotion, and prevention of hate violence victims. However, political actions are necessary to avoid the lack of knowledge and lack of training and professional tools that are widespread among healthcare staff. Therefore, the training of professionals and the establishment of specific protocols for action against hate violence would improve the care and long-term monitoring of victims, and the implementation of an epidemiological registry and surveillance system of hate violence would improve the care and prevention of hate violence in Spain.

## 1. Introduction

The World Health Organization (WHO) considers violence to be a global public health problem and has underlined the importance of the health sector’s approach for showing that victims of any type of violence require health and social healthcare, and that physical or psychological consequences can be maintained and developed in the medium and long term [1]. Recently, studies have been interested in a specific type of violence, namely hate violence, which is defined as the intentional use of force or physical power motivated by rejection and discrimination towards a characteristic, be it real or perceived, of the victim, such as their religious confession, ethnic or national origin, sexual orientation or gender identity, gender, age, disability, or homelessness, and which has a negative impact on the physical or mental health of the person attacked in the short, medium, or long term [2].

The Organization for Security and Cooperation in Europe (OSCE) reaffirms the commitment to promoting tolerance and combating discrimination “against any manifestation of aggressive nationalism, racism, patriotism, xenophobia, anti-Semitism, or violent extremism in any of the participants States, as well as any discrimination based on race, colour, sex, language, religion or belief, political opinions or other nature opinions, or based on origin, wealth, birth, or any other consideration” [2]. In this way, it urges member states to develop and maintain registers of crimes motivated by prejudice, as well as to report on existing legislation on crimes motivated by intolerance and discrimination.

Subsequently, in [3], participating states are again requested to compile and diffuse statistical data related to hate crimes, the enactment (where appropriate) of specific laws to combat this type of crime, the study of ways to provide advice and assistance to hate crime victims (and encourage their reporting), as well as to raise awareness and carry out educational initiatives for groups involved in caring for victims of hate crimes.

A Council Framework Decision [4] on combating certain forms and expressions of racism and xenophobia through criminal law established a common objective for criminal responses to this phenomenon and was an important milestone in the recognition of hate crimes at the European level. The law defines “hatred” as that based on “race, colour, religion, descent or national or ethnic origin”. However, it leaves it up to the member states to extend its scope to “offences against groups of persons defined according to other criteria (...), such as social status or political convictions”. The transposition into the Spanish context involved reforming the Criminal Code in 2015 [5].

Thus, Article 510 of the Spanish Penal Code refers to hatred, hostility, discrimination, or violence against people for reasons of belonging to a group, “for racist, anti-Semitic or other reasons related to ideology, religion or beliefs, family situation, belonging to an ethnic group, race or nation by their members, their national origin, their sex, orientation or sexual identity, for reasons of gender, illness or disability”, thereby extending the assumptions included in the European legislation.

In order to generate a multidisciplinary framework in which to combine preventive, investigative, and assistive actions for victims of hate crimes, the Action Plan to Combat Hate Crimes was adopted in Spain in 2019 [6], aimed at establishing measures for raising awareness, training, and care and response to this type of crime by the State Security Forces and Corps. Since its publication, the Ministry of the Interior has developed various protocols and guidelines for action on this issue, for example, the Protocol for Combating Illegal Online Hate Speech (2020) [7], the Good Practice Guide for Reporting Hate Crimes (2020) [8], and the Protocol for Action by the Security Forces for Hate Crimes and Conduct that Violates the Legal Rules on Discrimination (2020) [9]. However, none of the instruments adopted are aimed at assisting the healthcare field with the care of victims, as they are limited to raising awareness and training law enforcement personnel. To the best of our knowledge, there is neither specific policy nor specific protocols for action against hate violence in healthcare systems in the different Autonomous Communities.

Information on the magnitude and prevalence of hate violence is insufficient and difficult to access [10]. In Spain, although the crime statistics [11] have specifically collected facts or investigations on hate crime since 2014 which makes it possible to access cases of violence that have been investigated as possible hate crimes by the security forces, they do not reflect all possible incidents of hate violence. With fluctuations, the annual evolution shows a growing prevalence, which has gone from 1285 (in 2014) to 1706 cases (in 2019), with the available data in the last two years showing an increase of 12.6% in 2018 and 6.8% in 2019. Concerning the Autonomous Communities in which this study was conducted, the statistics on hate crimes by the Ministry of the Interior in 2019 showed that Catalonia had the highest incidence of known incidents (30.1% of the Spanish total), followed by the Community of Madrid, with 15.1%. In contrast to these two Communities, Castilla-La Mancha in 2019 had an incidence of hate crimes of 2.9% compared with the national territory as a whole.

The Ministry of Interior [11] has classified hate crimes into the following areas: racism/xenophobia, sexual orientation and gender identity, religious beliefs or practices, anti-Semitism, disability, aporophobia, ideology, and gender. In addition, since 2018, discrimination based on illness, generational discrimination, and, at the end of 2018, anti-Gypsyism have been included as categories. The most common typology recorded in Spanish crime statistics in 2014 was offences related to sexual orientation and gender identity (513 known facts); between 2015 and 2017, the highest number of offences was for reasons of racism and xenophobia (505 and 416, respectively, and 524 known facts). Since 2018, the ideology of the victim has been the reason for the highest number of offences committed (596 in 2018 and 615 in 2019). This is not in line with the population’s perception of the most frequent forms of discrimination in Spain [12], which, in order, are against the gypsy population (65%, 61% European average), transgender people (58%, 48% European average), skin colour (55%, 59% European average), sexual orientation, and ethnic origin (54% in both cases). Moreover, a comparison among other countries is difficult because the methodology, concepts, and categories used in each country are very heterogeneous. For example, in the USA, of the 6121 hate crime incidents in 2017, 20.8% involved religious prejudice, 19.6% involved sexual orientation and gender identity, and 57% involved incidents focused on racial, ethnic, and other descent groups [13]. However, what is consistent in the analysis is that the magnitude of hate crime is widely underestimated [13].

As the most extreme consequence, it has been established that hate crimes that resulted in death committed in the Spanish State between 1990 and 2020 reached 101 cases with 103 fatalities [14]; however, the prevalence of hate violence may have been underestimated, since it has been estimated that only 4.3% [14], or between 10 and 20% of victims report hate violence [15].

For the most part, the most immediate consequence of hate violence is embodied in physical injuries; in fact, the crime statistics collected on hate crimes in Spain [11] for criminal offences indicate there were injuries, on average, in 20% of cases (21.9% in 2019). The psychological sequelae that occur in the long term must also be considered and commonly involve fear, isolation, sleep disturbance, increased alarm, traumatic stress, anxiety, and anguish. In addition, hate crimes have a broad community impact [16], since they transmit a threatening message to groups of victims, which deepens their fear, their powerlessness, and the low reporting of these crimes. This is precisely the goal of considering hate violence to be a public health problem, since it deals with the health of the groups most at risk from the perspective of improving or promoting health and preventing recurrence of diseases or injuries.

A cross-sectional study of a series of cases of aggression treated in the emergency rooms of two hospitals in Madrid (Spain) [17] found that the most frequent motivations were physical appearance, nationality, and ethnic origin. Aggressive actions that occurred in the streets (50%) were perpetrated by more than one person (61%) and by men (83%) for the most part. The most common injuries were contusions (71%), the most frequent locations were the head and neck (71%), and only a few cases required admission to hospital (8%) or were repeat assaults. The victims were men (61%), Spanish (50%), with high-middle injuries with mild prognoses (80%). In addition, it was found that people of foreign origin were more frequently attacked for reasons of hatred (50% versus 32%) than for other motivations, being for the most part Latin Americans (50%), who have been identified in other international studies [18] that also detected greater severity and aggression rates in foreigners. This study found that victims indicated that they were willing to report the event (90%), but the literature has consistently indicated that there are obstacles to filing a complaint such as lack of knowledge or information, fear of consequences, lack of trust in agencies, and accessibility [19].

Health services play a major role in tackling hate violence, usually by providing emergency services, although, beyond the treatment of immediate physical injuries, there have been questions concerning whether they adequately identify cases of hate violence, or if they are the most appropriate service for assistance [17,20]. Health services must guarantee victims receive comprehensive care, including appropriate diagnoses and treatment, as well as a risk assessment of further aggression and, if needed, possible referral to other resources [10]. The continuity of care from primary care and mental health is also a crucial factor for a victim’s recovery and the prevention of cases of violence [15].

As far as we know, there are no international studies on the perceptions of health personnel on hate violence. The objectives of this study include perceptions of hate violence, knowledge of hate violence, the capacity to detect cases, and the existence of tools and resources necessary for the optimal treatment of hate violence in health services. The aim is to identify the elements that would allow health services to improve the care provided to victims and their contribution to clarifying the real magnitude of the phenomenon.

Therefore, based on the perceptions of healthcare personnel regarding hate violence, which has a clear impact on people’s health, the aim of this study is to identify possible areas for improvement in healthcare and health prevention with respect to this type of violence. In addition, we aim to describe the characteristics of care for victims of hate violence in Spain to detect potential improvements in the identification and correct classification of victims and hate crime violence. By establishing an epidemiological registry and surveillance system of the registration of hate violence, an increase in the number of reports would provide a more realistic indication of the magnitude of the phenomenon for more comprehensive care of victims, as well as strategies for the prevention of hate violence.

## 2. Materials and Methods

### 2.1. Design and Instrument

An exploratory study with a descriptive, observational, and cross-sectional design was conducted through a specific questionnaire that was developed to determine the main perceptions of health professionals of hate violence. The design of the questionnaire began with a review of the literature for the selection of content and items and it was revised by an expert group to evaluate the validity of the content and the relevance of the items. Then, a pretest was conducted to correct the items that were not properly understood or that generated uncertainty in the answers. Finally, 27 items were selected, in addition to sociodemographic data (sex, age, nationality, marital status, educational level). The questionnaire was structured by content as follows: location of the professional (type of professional, work service, time of professional practice, and educational level); perceptions of the most common discriminations (areas of discrimination were as collected in the Eurobarometer [12], for example, they were asked to indicate the reasons for the most frequent hate violence in their service in the following areas: ethnic origin, gender, gender identity, sexual orientation, age, religion or belief, disability, older people, and homeless people, where examples were given in each case, such as in sexual identity, i.e., being gay or lesbian); training in hate violence (training and knowledge about hate violence, acquisition of training, and existence of protocols and a definition of hate violence in the service of work); frequency of cases of hate violence (cases attended, frequency, scope of violence, ex officio complaint, and data recorded in a report); characteristics of the cases attended (complaint, time elapsed, referral, places, injuries attended, and consequences for victims); and perceptions of care from health services (detection capacity by health services and possible improvements in care from health services). The assessment of the items, where appropriate, was established on a Likert-type scale (1–4).

### 2.2. Sample

An incidental non-probabilistic sample (*N* = 199) was used to collect data from health professionals (nursing 45.2%, medicine 44.2%, emergency telephone service 3%, social work 2.5%, and others 5%) in three Autonomous Communities (52% Castilla-La Mancha, 25% Catalonia, and 23% Madrid) and three healthcare facilities (49.5% primary care, 41.3% hospital emergencies, and 9.2% emergency hotline/ambulance). The opportunity to carry out a randomized study by conglomerates was explored, which also represented the territorial distribution of the services in the three Autonomous Communities; however, given the difficulties due to the lack of permits to access some health services, the exploratory nature of the study, as well as the timeframes and resources of the study, it was decided to carry out incidental sampling to facilitate access to the widest possible sample. The health authorities were contacted, and permission was requested to later contact those responsible for emergency services, hospital emergencies, and primary care in the three Autonomous Communities that agreed to contribute. The questionnaires were carried out by those responsible for the study in the hospital services, and all professionals who volunteered to participate in the study were surveyed. The average age of the people surveyed was 44.6 years with an age range of 23–67 years; the age distribution was concentrated in the middle (71% between 31 and 55 years). They had an average professional experience of 19.9 years, with a range between 1 and 43 years. The sociodemographic characteristics of the sample are described in Table 1.

### 2.3. Data Analysis

A descriptive analysis involved calculating the distribution of absolute and relative frequencies of variables, using descriptive statistics and contingency tables. Statistically significant differences were verified using the chi-square test (X^2^), for a value of *p* ≤ 0.05, to check the differences in knowledge about hate violence, the existence of specific attention protocols, and the frequency of attention to cases by Autonomous Communities and assistance services. A lack of response or NA/NK (no answers/not know) were considered to be missing values. The statistical package SPSS v24 (IBM, New York, NY, USA) was used.

### 2.4. Ethical Considerations

The design and questionnaire were approved by the Ethics Committee of the General Hospital “Virgen del Prado” in Talavera de la Reina (Health Service, Castilla-La Mancha, Spain). Participants were previously asked for informed consent, a mechanism to challenge their participation.

## 3. Results

### 3.1. Knowledge and Training on Hate Violence

The health personnel who were surveyed expressed (see Table 2) very low knowledge (77.9%) of hate violence; only 17.6% indicated very high knowledge, 80.4% did not have training on hate violence, and only 19.6% indicated having specific training, without finding significance by types of professionals in relation to specific training on hate violence (X^2^ = 0.451, *p* = 0.798).

Regarding the differences in specific training by Autonomous Communities, there were no significant differences (X^2^ = 2082, *p* = 0.353), although the Community of Madrid had a higher percentage of trained professionals than Catalonia or Castilla-La Mancha (see Table 3).

### 3.2. Concept of Hate Violence and Action Protocols in Health Services

Among the health personnel, 80.5% indicated that they did not have a common definition of hate violence in their services (see Table 4), although there were no significant differences among types of professionals (X^2^ = 4.296, *p* = 0.367) or Autonomous Communities (X^2^ = 3.727, *p* = 0.155).

Seventy-five per cent of the sample explicitly indicated non-existence in their service of a specific action protocol for hate violence (see Table 5), which was greater in primary care than in hospital emergencies and emergency hotlines/ambulances, although without significance in the differences (X^2^ = 2.382, *p* = 0.304). The differences in the existence of a specific action protocol by Autonomous Communities are significant (X^2^ = 6.831, *p* = 0.033), since, in Catalonia, 37.8% of professionals said they have this protocol in their service; in Castilla-La Mancha, this drops to 22%, and in Madrid, it drops to 15.2%. Participants indicated that they did not have a common definition of hate violence in their services, although there were no significant differences between types of professionals (X^2^ = 4.296, *p* = 0.367) or Autonomous Communities (X^2^ = 3.727, *p* = 0.155).

In addition, we investigated which elements could influence a higher prevalence of ex officio complaints from the health services, such as the training of professionals, knowledge about hate violence, the existence of specific care protocols, or the existence of a common definition in the service. The existence of specific care protocols is significant in relation to the presentation of complaints from the service (X^2^ = 11,047, *p* = 0.001), as well as training on hate violence (X^2^ = 4180, *p* = 0.041) and the knowledge they claim to have about hate violence (X^2^ = 6.030, *p* = 0.049). However, the existence of a common definition in the service is not significant in relation to the complaints filed from the performance service.

### 3.3. Periodicity and Frequency of Attention to Cases of Hate Violence

The participants were asked about the cases attended in the last 12 months (see Table 6); 81.2% indicated having attended a case in the last 12 months (between 1 and 10), 12.1% between 11 and 20 cases, 3.0% between 21 and 30 cases, and 3.6% more than 30 cases. They indicated that they did not have a common definition of hate violence in their services, although there were no significant differences between types of professionals (X^2^ = 4.296, *p* = 0.367) or Autonomous Communities (X^2^ = 3.727, *p* = 0.155).

The prevalence of cases attended in the last 12 months by services was significant (X^2^ = 21.120, *p* = 0.002). If we combine the four intervals of cases attended to into two (i.e., 1–20 and more than 20 cases), 20% of the hospital emergency hotline/ambulance personnel attended “more than twenty cases”, 8.20% of the hospital emergency personnel, and only 2.7% of primary care. However, in the range of 1–20 cases, the order of services is reversed, 97.3% of primary care personnel attended between 1 and 20 cases in the last 12 months, 91.8% of hospital emergency personnel, and 80% of emergency hotline/ambulance personnel.

The differences in the prevalence of attention to hate violence cases by Autonomous Communities are significant (X^2^ = 4.353, *p* = 0.629), with around 80% of the professionals who attended between 1 and 10 cases in the last 12 months; the differences between medical and nursing personnel are not significant (X^2^ = 1.004, *p* = 0.800).

### 3.4. Reasons, Complaints, Injuries, and Effectiveness in Detection

In the sample, 58.3% of the participants indicated that their service denounced cases of hate violence ex officio, while 21.6% indicated that it did not (20.1% NA/NK). By Autonomous Communities, the differences are significant (X^2^ = 20.997, *p* = 0.000). The data for Catalonia (81.4%) and Castilla-La Mancha (81.8%) are similar; in Madrid, only 43.2% indicated that their service denounced cases ex officio.

Regarding the most common physical injuries that they attended, 81.5% were acute physical injuries, 16% long-term pathologies related to a poor perception of health, and 2.5% serious dangerous injuries. Regarding the most common mental injuries, 33.8% considered that there was “impotence and defencelessness”, 28.4% “extreme fear” and 27.7% “stress and anxiety”.

Fifty-six percent of the health personnel considered that the effectiveness of the health services for detecting hate violence was “little or null”, 37.7% “medium”, and 6.3% “high”; they indicated that the main improvement measures were: training of professionals (50%), creation of protocols (26.3%), coordination between public services (13.8%), and awareness of health professionals (8.6%).

## 4. Discussion

There are few studies on hate violence in the field of healthcare in Spain, as well as in the rest of the world, with the exception of the SIVIVO initiative (surveillance system against hate violence) in Spain, which has highlighted the importance of the role of the health sector in addressing hate incidents, the development of a detection questionnaire [20], and the analysis of cases in another study treated in an emergency room [17]. However, to the best of our knowledge, the perceptions of healthcare personnel caring for victims of hate violence, so far, have not been explored.

Our results show that the care of hate violence victims in healthcare services in Spain is quite widespread, since 82.4% of the staff attended some case (1–20 cases) in the last 12 months of their professional performance. Nevertheless, the lack of training (80.4%) and knowledge (77.9%) of health personnel on hate violence are generalized, and therefore the differences by types of professionals, services, or Autonomous Communities are minimal, which explains the non-existence of relationships with significant statistics. The minimal differences found, for example, may be due to the prevalence of cases attended, the higher prevalence in the most populated communities, or the sensitivity of professionals and not to the existence in any Community of specific institutionalized protocols for the care of victims of hate violence.

In addition, this widespread lack of knowledge is corroborated by the results of the health professionals’ responses who indicated that, in their services, there was no common definition of hate violence (80.5%) or action protocols (75%) in the case of hate violence. There were no significant relationships by services, that is, there were no protocols and a common definition was similarly lacking and generalized in all cases. However, the differences by Autonomous Communities were significant, and in Catalonia (37.8%) more services had a specific protocol than in Castilla-La Mancha (22.0%) or Madrid (15.2%). Consequently, we consider that the differences between Communities may be due to the prevalence of cases attended in each specific service or to the responsiveness of their professionals, but not to the existence of institutionalized tools or protocols for the care of victims of hate violence in any Autonomous Community.

Eight out of ten professionals indicated that they had attended a case of hate violence in the last 12 months (between 1 and 10 cases), although the majority perceived that they hardly ever or never attended hate violence situations (85.6%). Regarding the prevalence of cases attended, the significant statistical differences (*p* = 0.002) indicate that, in the highest range of incidence of cases (more than twenty cases), emergency hotline/ambulance services attended more cases, followed by hospital emergency services and primary care services. This has a certain logic, since the attacks detected may be attended to, in the first instance, by the emergency hotline/ambulance services, since they are the first to respond, which can be subsequently referred to the hospital emergency services, in line with the places where hate violence occurs most frequently (49.3% at home and 40% public road), according to health personnel.

Acute physical injuries (81.5%) are adequately cared for, especially in the emergency hotline/ambulance services, immediately after the incident (63.5%), or after one hour (23.3%); however, the pathologies related to a poor perception of the state of health (16%), extreme fear (28.4%), or stress and anxiety (27.7%) are perceived to a lesser extent. This indicates high efficiency in the treatment of physical injuries, but that the care and monitoring of potential psychic pathologies of the victims can be improved.

Therefore, 56% of health personnel considered the effectiveness of health services for detecting hate violence to be “little or null”, and they proposed the following actions for increasing their capacity to detect hate violence: training of professionals (50%), specific protocols (26.3%), and coordination between public services (13.8%). Their perceptions agree with our results; therefore, the presentation of ex officio complaints from the health services has been related to the training and knowledge of health personnel, as well as to the existence of specific protocols for action against hate violence in health services. In addition, the recommendations of organizations such as the OSCE [2,3] are in line with this, but in Spain the only protocols adopted to date are specifically for the State Security Forces.

Consequently, the role of health services in hate violence is essential, since our results show that health personnel are among the first to interact with victims, who quickly go to health services by themselves (39%) or are referred from hospital emergencies (20.8%) and, even when they are referred by the police (30.8%), it is still health professionals who treat the possible pathologies or sequelae. Taking this into account, in addition to the fact that in the opinion of health personnel (77.3%), victims “almost never or never” report, health services constitute “the gateway” to the care and possible appropriate guidance of victims of hate violence; therefore, they are cardinal in the quantification, detection, care, and monitoring of victims of hate violence.

Simultaneously, our results on the perceptions of health personnel seem to indicate an underdiagnosis of hate violence, which, according to other studies, leads to underreporting by victims, with a lack of knowledge trust in institutions, as well as accessibility difficulties as the main barriers to doing so [19]. Increased training and implementation of specific protocols [15] would contribute to an increase in complaints, and also to improving coordination, guidance, and treatment in the long term, which, together with the implementation of a systematic and standardized collection system of data and statistics on hate violence, would result in substantial qualitative improvements in the treatment, promotion and prevention of hate violence.

## 5. Conclusions

The physical injuries of hate violence victims are effectively treated by health services in Spain in the different Autonomous Communities analysed. However, it has been questioned whether the long-term care and follow-up of victims are adequate, given coordination difficulties and the lack of professional tools (training, specific protocols, and specific registries) and specific resources for victims.

Therefore, hate violence may be underdiagnosed in the Spanish health system because of generalizations by health staff, due to lack of knowledge, training, action protocols, systematic registration, and long-term follow-up of victims. In addition, our results show that training, knowledge, and the existence of specific protocols for hate violence are associated with the presentation of ex officio complaints by the health services, which would contribute to an increase in ex officio complaints by the health services, and therefore stimulate the establishment of an adequate registry of cases and their circumstances, epidemiological follow-up, and the establishment of the real magnitude of the phenomenon of hate violence.

Consequently, the potential of health services is being wasted, as a “gateway” for victims of hate violence to public social protection systems, for detecting the magnitude, characterization, and prevention of hate violence. Thus, improvements in health policies on hate violence in Spain are necessary for the establishment of common and homogeneous action protocols in the different Autonomous Communities, specific training on hate violence for health personnel, and a system of registry and epidemiological surveillance of hate violence in the Spanish health system are required.

The main limitations of this study are the non-probabilistic sample that does not allow the results to be generalized, and the cross-sectional nature of the design that prevents understanding of the causality of the associations found, and therefore should be considered with caution.

## Figures and Tables

**Table 1:** Sample description.

		*N* (199)	%
Sex (*n* = 197)	Male	62	31.5
	Female	135	68.5
Nationality (*n* = 194)	Spanish	194	98.5
	Other	3	1.5
Marital status (*n* = 193)	Married	107	55.4
	Single	53	27.5
	Widowed	4	2.1
	Cohabitation	29	15.0
Educational level (*n* = 199)	Secondary education	2	1.0
	Tertiary education (short cycle)	2	1.0
	Grade	82	41.2
	Master’s degree	94	47.2
	Doctorate degree	19	9.5
Professions (*n* = 199)	Medicine	88	44.2
	Nursing	90	45.2
	Phone operator	6	3.0
	Social Worker	5	2.5
	Other	10	5.0
Service (*n* = 196)	Primary care	97	49.5
	Hospital emergencies	81	41.3
	Emergency hotline/ambulance	18	9.2
Autonomous Community (*n* = 196)	Catalonia	45	23.0
	Castilla-La Mancha	102	52.0
	Madrid	49	25.0

**Table 2:** Training on hate violence by health profession, column percentages.

	Yes		No	
	*n*	%	*n*	%
Nursing	17	44.7%	70	44.9%
Medicine	16	42.1%	71	45.5%
Other	5	13.2%	15	9.6%

**Table 3:** Specific training by Autonomous Communities, row percentages.

	Yes		No	
	*n*	%	*n*	%
Catalonia	8	17.8%	37	82.2%
Castilla-La Mancha	17	17.3%	81	82.7%
Madrid	13	27.1%	35	72.9%

**Table 4:** Common definition of hate violence in the services by profession and Autonomous Communities, column percentages.

	Medicine		Nursing		Other		Total	
	*n*	%	*n*	%	*n*	%	*N*	%
Yes	14	16.1%	18	20.5%	6	30%	38	19.5%
No	73	83.9%	70	79.5%	14	70%	157	80.5%
	**Catalonia**		**Castilla-La Mancha**		**Madrid**		**Total**	
	***n***	**%**	***n***	**%**	***n***	**%**	***N***	**%**
Yes	12	26.7%	19	18.8%	5	10.9%	36	18.8%
No	33	73.3%	82	81.2%	41	89.1%	156	81.3%

**Table 5:** Existence of a specific protocol on hate violence by services, column percentages.

	Hospital Emergencies		Emergency Hotline/Ambulance		Primary Care		Total	
	*n*	%	*n*	%	*n*	%	*n*	%
Yes	24	30.0%	5	29.4%	19	20.2%	48	25.1%
No	56	70.0%	12	70.6%	75	79.8%	143	74.9%
Total	80	100%	17	100%	94	100%	191	100%

**Table 6:** Prevalence of hate violence cases attended by services, column percentages.

	Hospital Emergencies	Emergency Hotline/Ambulance	Primary Care	Total
Between 1 and 10	83.6%	60.0%	82.4%	82.4%
Between 11 and 20	8.2%	20.0%	14.9%	14.9%
Between 21 and 30	6.8%	0.0%	0.0%	0.0%
More than 30	1.4%	20.0%	2.7%	2.7%
Total	100%	100%	100%	100%
